# Automating the Timed Up and Go Test Using a Depth Camera

**DOI:** 10.3390/s18010014

**Published:** 2017-12-22

**Authors:** Amandine Dubois, Titus Bihl, Jean-Pierre Bresciani

**Affiliations:** 1 Department of Medicine, University of Fribourg, 1700 Fribourg, Switzerland; jean-pierre.bresciani@unifr.ch; 2 Cantonal Hospital, 1700 Fribourg, Switzerland; Titus.Bihl@h-fr.ch

**Keywords:** timed up and go, automated clinical test, objective assessment, elderly people, depth camera, fall prevention

## Abstract

Fall prevention is a human, economic and social issue. The Timed Up and Go (TUG) test is widely used to identify individuals with a high fall risk. However, this test has been criticized because its “diagnostic” is too dependent on the conditions in which it is performed and on the healthcare professionals running it. We used the Microsoft Kinect ambient sensor to automate this test in order to reduce the subjectivity of outcome measures and to provide additional information about patient performance. Each phase of the TUG test was automatically identified from the depth images of the Kinect. Our algorithms accurately measured and assessed the elements usually measured by healthcare professionals. Specifically, average TUG test durations provided by our system differed by only 0.001 s from those measured by clinicians. In addition, our system automatically extracted several additional parameters that allowed us to accurately discriminate low and high fall risk individuals. These additional parameters notably related to the gait and turn pattern, the sitting position and the duration of each phase. Coupling our algorithms to the Kinect ambient sensor can therefore reliably be used to automate the TUG test and perform a more objective, robust and detailed assessment of fall risk.

## 1. Introduction

Falls in elderly people often result in fractures, trauma, disabilities, and loss of activity. In fact, falls are the second biggest cause of death reported in the world (WHO [1]). Therefore, fall prevention constitutes a major human and economic issue, nowadays and for years to come. The Timed Up and Go test (TUG test) is widely used by healthcare professionals to assess fall risk and distinguish high vs. low fall risk individuals. It is recommended by both the American Geriatrics Society and the British Geriatric Society [2]. The TUG is based on the Get Up and Go (GUG) test, originally proposed by Mathias et al. [3]. In the GUG test, healthcare professionals observe a person who stands up from a chair, walks 3 m, turns 180°, walks back to the chair and sits down. Then, the healthcare professionals evaluate the person on a five-point ordinal scale : “normal”, “very slightly abnormal”, “mildly abnormal”, “moderately abnormal” and “severely abnormal”. The TUG test is the timed version of the GUG test. It was introduced by Podsiadlo and Richardson [4] to diminish the subjective nature of scoring. Physicians usually use a stopwatch to time the test, from stand up to sit down. Several studies [5,6,7] highlighted a correlation between the time required to perform the TUG test and fall risk in elderly people, a slower performance on the test being associated with a higher fall risk.

Though the TUG test is widely used by healthcare professionals, notably because of its simplicity and the ease with which it can be performed in clinical environments, some authors criticized it [8,9]. In particular, the TUG test is considered as being too dependent on the clinicians conducting it as well as on the environmental conditions in which it is conducted. When looking at the literature and at clinical practice, the threshold used to discriminate low and high fall risk individuals can vary from 10 s to 25 s. For instance, Shumway-Cook et al. [5] have proposed a cut-off of 13.5 s to discriminate low and high fall risk individuals. Along a similar line, Dite and Temple [10] found that a cut-off value of 13 s resulted in a discrimination sensitivity of 89% and a discrimination specificity of 93%. On the other hand, Okumiya et al. [11] suggested that a cut-off of 16 s constituted a good predictor of fall risk. The environmental conditions chosen by the clinicians can also increase the variability of the test: for instance, the 3 m of walking are not always respected. In addition, a chair without arms is sometimes used. Finally, the instructions given and the test evaluation can vary depending on the experience of the medical staff. For instance, the instruction to walk as fast as possible or as comfortably as possible can result in time differences. To limit such biases, an evaluation grid including several ‘assessment observations’ is sometimes used by healthcare professionals. This grid can then be used to determine a score on the five-point scale proposed by Mathias and colleagues [3]. However, the assessment observations can slightly differ between institutions. In addition, healthcare professionals are not always aware of the existence of this grid, and it is seldom used in practice.

Another limitation of the TUG test is that, currently, it is almost exclusively run by specialized clinicians. We believe that, if such a test could be performed routinely and automatically by other healthcare professionals, notably by general practitioners, fall prevention could be greatly improved. Specifically, new technological solutions could be adopted to help perform automatic and more objective clinical assessments. In line with this, instrumenting clinical assessments using technology is an issue addressed by a growing number of research groups. The aim is to build a system (based on sensors such as video or accelerometers) automatically providing data relative to the performed test. In their review of 2015, Sprint et al. [12] listed the different techniques to automate the TUG test. Weiss et al. [13] automated the TUG test using accelerometers, whereas Salarian et al. [14] combined accelerometers and gyroscopes. The use of sensors allowed these authors to discriminate healthy subjects from Parkinson’s disease patients, which was otherwise impossible using the traditional TUG assessment only. Beyea et al. [15] used the inertial measurement units (accelerometers, gyroscopes and magnetometers) with healthy adults aged 21 to 64 years old to detect TUG phases (sit-to-stand, walk, turn, stand-to-sit). They validated the correlation between the TUG phase durations obtained with their sensors and those measured with an optoelectronic motion capture system. Accelerometers have also been used with the TUG test to automatically classify high and low fall risk individuals [16,17,18]. Whereas traditional TUG assessments are mostly based on the time required to perform the test, these studies included additional parameters/criteria to discriminate high fall risk from low fall risk individuals.

The TUG test can also be “automated” using video recording, which has the advantage not to interfere with the tested person. For instance, Skrba et al. [19] used two webcams to extract different parameters such as the total walking time, the number of steps, the stability into and out of the turn, etc. They concluded that the walk duration and the time between turning and sitting back on the chair were the most relevant parameters to classify low and high fall risk individuals. Other researchers used video to analyze a specific component of the test. For example, Wang et al. [20] focused on the turn phase, whereas Frenken et al. [21] studied the walking phase. Lohmann et al. [22] used a Kinect camera with skeleton tracking to automate the test. They detected events, as starts moving, ends uprising, starts walking, starts rotating, etc., and compared these values with manual labeling. Kitsunezaki et al. [23] performed a study similar to that of Lohmann et al. but they used three Microsoft Kinect cameras placed in front of, to the side of and above the chair, in order to determine the position of the Kinect, which minimized timing errors. They concluded that placing the Kinect 4 m in front of the chair is the configuration giving the most accurate data.

We developed a system that automatically discriminates low vs. high fall risk individuals. This system measures the total time required to complete the test, as healthcare professionals do, but, in addition, it measures complementary parameters, such as time to sit down, time to walk, spatio-temporal parameters of gait, etc. These additional parameters cannot be measured with the traditional TUG test and they provide supplementary qualitative information that can help the clinician identify which aspects/components of the test are problematic for the tested person (i.e., walking, turning, sitting or getting up). In that respect, measuring these parameters constitutes an automatic variant of the evaluation grid. However, the automatic assessment of these parameters with our system presents two main advantages over the “traditional” evaluation grid. First, because it is built in the system, the assessment takes place every time the test is performed, whereas as mentioned above, the evaluation grid is seldom used by clinicians. Second, these parameters provide a more objective evaluation than the grid, the results of which often depend on the clinician performing the evaluation.

For the hardware, our system relies on the Microsoft Kinect v2, that is low cost, easy to use, and works with the silhouette (i.e., using depth points only), which is of less privacy concern [24]. Most studies based on the Kinect sensor use the Microsoft Software Development Kit (SDK). Our system does not rely on the Microsoft SDK but instead uses an algorithm developed by Dubois and Charpillet [25]. This is because, with our system, the parameters are extracted from the vertical displacement of the geometric center of the body, so that an accurate representation of the skeleton and the body segments is not necessary. Our approach has two main advantages. First, parameters can be extracted even if the feet of the walking person are occluded. Second, the performance of the analysis is relatively unaffected by the angle of view of the sensor, which also constitutes a major advantage for monitoring individuals in any place, and especially in furnished environments (e.g., physician’s office, patient’s room). In this study, the same sequences of the TUG test were evaluated both by our algorithm and by healthcare specialists. First, we tested whether the measurements of test duration made with our system matched those made by healthcare professionals. For that, we used statistical analyses to compare the test durations automatically extracted with the Kinect sensor to those measured by healthcare specialists. In addition, we assessed which test parameters are the most relevant to classify individuals into high or low fall risk population. Finally, we used machine learning methods to determine whether our system can constitute an automatic and objective alternative to the evaluation grid. Our aim was to build a tool that consistently provides the same values regardless of the clinician conducting the test and of the place where it is performed.

## 2. Methods

### 2.1. Participants

Thirty-seven participants (23 females, 14 males) aged 61 to 93 (mean = 83.63) were evaluated with the TUG test. They were all patients residing in a transitional care unit. The main inclusion criteria were being aged 60 to 95 years old, having a frailty level between 4 and 7 as determined by the FRAILTY scale, and being able to walk at least 10 meters straight. Individuals suffering from severe dementia (with comprehension difficulty such as aphasia or alexia), visual disorders (e.g., advanced macular degeneration, blindness), or having a questionable cardio-pulmonary status (e.g., cardiac failure, pulmonary embolism, oxygen therapy) were excluded. On the other hand, individuals using auxiliary means to ambulate were included, except if they required a wheelchair. Fall history was not included as an inclusion/exclusion criterion. All participants signed an informed consent and the study was approved by the local ethics committee.

### 2.2. Data Acquisition

**Figure 1:**
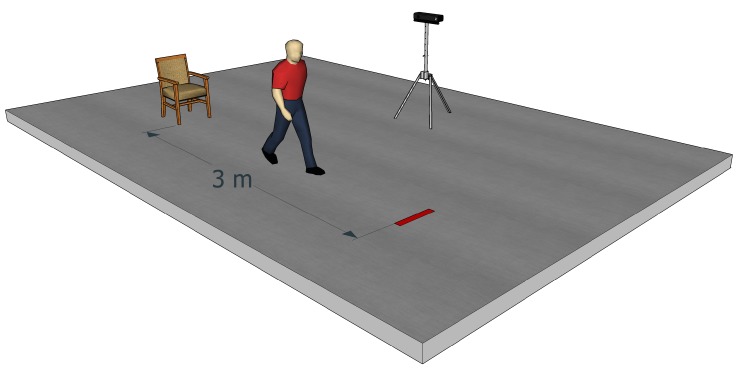
Timed Up and Go test: at the signal of the clinician, the participant has to get up, walk 3 m to the red mark, return to the chair and sit down.

A standard chair with arms was used. A mark was placed on the ground 3 m in front of the chair to indicate to the participants the place where they had to turn around. At the beginning of the test, participants were sitting on the chair. After a signal given by the clinical staff, the participant had to get up from the chair, walk, turn around after passing the mark located on the ground, go back and sit on the chair (see Figure 1). The participants were instructed to walk at a normal and comfortable speed. The participants walked perpendicularly to and at a distance of 4 m from the Kinect sensor. This sensor recorded the movement pattern of the participants. Two clinicians timed with a stopwatch the execution of the test (for the 37 participants) and also completed an evaluation grid comprising different assessment criteria (for 34 participants only). Eighteen participants were “evaluated” by a clinician having five years of experience, and the remaining nineteen participants by a clinician having four years of experience. The evaluation grid was divided into four evaluation criteria associated with several score items. The first criterion evaluated by the clinical staff was the transition from sitting to standing (Did the person need several trials? Did he/she lean on the chair’s arms to get up? Did he/she have a correct posture?). The second criterion was the gait pattern (Did the person walk straight normally?). The third criterion was the turn execution speed. The last criterion was the quality of the transition from standing to sitting. The completion of this evaluation grid allows clinicians to classify participants into three groups. Individuals obtaining a score of 0 are considered as “fall risk free”. Individuals obtaining a score of −1 or −2 are considered as “moderate risk of fall”. Individuals obtaining a score inferior to −3 are considered as “high risk of fall, requiring inclusion in a rehabilitation program for the lower limbs”. Each participant performed the TUG test up to three times (depending on their physical condition), with a 3–5 min rest interval between each test. Three participants performed the test one time only, and six participants performed the test two times only.

### 2.3. Preprocessing

**Figure 2:**
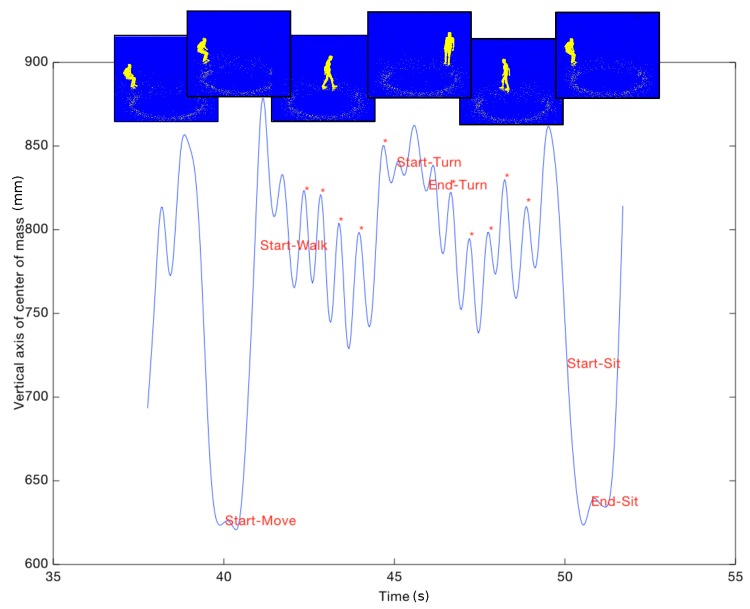
Trajectory of the centroid of a participant and automatic detection of six events.

**Figure 3:**
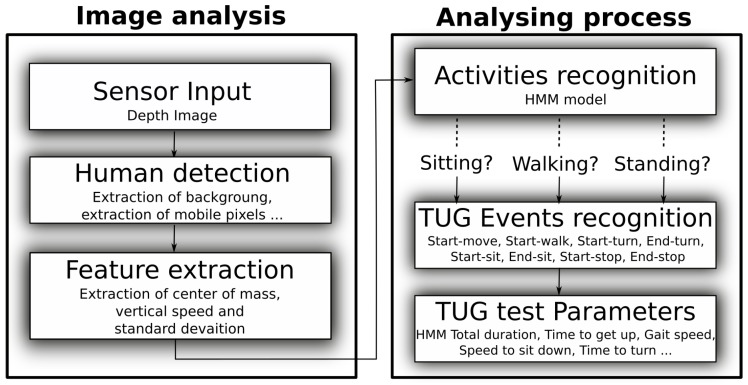
Automatic detection method of the different events of the TUG test.

Our processing method was only based on the depth images provided by the Kinect sensor, and the silhouette of the individuals was extracted using the background substraction method presented in Dubois and Charpillet [26]. First, the centroid of the person was computed. This centroid corresponded to the average of all points belonging to the silhouette. An algorithm using a Hidden Markov Model (HMM) was then used to analyse the trajectory of the centroid along the vertical axis. This algorithm identified the activity an individual was engaged in, such as walking, sitting, standing, etc. [26]. Here, we used the activity recognition algorithm to identify six different events: start-move, start-walk, start-turn, end-turn, start-sit, end-sit (see Figure 2). The “start-move” event corresponded to the first frame in which the algorithm identified that the sitting participant started a new activity. The “start-walk” event was identified from the beginning of the HMM activity “walking”. “Start-sit” and “end-sit” identified the beginning and the end of the sit down action and were extracted from the descent transition between the HMM activities “walking” and “sitting”. “Start-sit” was detected as the first frame following the end of HMM activity “walking” and “end-sit” as the last frame before activity “sitting”. To detect the turn, we extracted the person’s position furthest from the chair and from this position we kept all points located beyond 95% of the maximum distance. Depending on the participants, two additional events could occur: start-stop and end-stop. The stop, corresponding to activity “stand”, was generally due to a hesitation about the following task to perform. The different steps of the processing algorithm are represented in Figure 3. 

### 2.4. TUG Test Derived Parameters

Parameters that we automatically extracted from the Kinect sensor were chosen based on the four evaluation criteria of the evaluation grid (walking, turning, sitting or getting up) to analyze the TUG test. Based on the six events extracted from depth images as described in Section 2.3, we selected the following parameters:

- total duration (s): time between “start-move” and “end-sit”;
- time to get up (s): time between “start-move” and “start-walk”;
- speed to get up (mm/s): maximum vertical upward speed between “start-move” and “start-walk”;
- time to sit down (s): time between “start-sit” and “end-sit”;
- speed to sit down (mm/s): maximum vertical downward speed between “start-sit” and “end-sit”;
- time to walk (s): time between “start-walk” and “start-turn” plus time between “end-turn” and “start-sit”. If the participant stopped during the walk, the time between events “start-stop” and “end-stop” was discarded from the calculation;
- number of stop: number of times that the participant hesitated;
- width of turn (cm): distance between the position at “start-turn” and “end-turn”;
- time of turn (s): time between “start-turn” and “end-turn”;
- greatest width between forward walk and return walk (cm): calculation of the greatest distance between the position of the participant in forward walk and return walk.

The vertical trajectory of the centroid allowed us to identify the steps of the person. Figure 2 shows the local maxima detected during the walk (indicated by asterisks). One step corresponds to the interval between two local maxima. Step lengths were estimated from the corresponding 3D coordinates of the centroid. This information allowed us to obtain the following additional TUG test parameters:

- number of steps;
- mean and median step length (cm): mean or median of the distance between two local maxima;
- Coefficient of Variation (CV) of mean step length (%): standard deviation of the step length divided by the mean step length;
- mean and median step duration (s): mean or median of the duration between two local maxima;
- CV of the mean step duration (%);
- mean and median cadence of walking (steps/min): cadence was calculated as one divided by the step duration, mean or median of cadence of the two walking sequences (forth and back);
- CV of the mean cadence (%);
- gait speed (during the two walking sequences, cm/s): sum of step lengths divided by the sum of step durations.

The algorithm used to determine the gait parameters is detailed in Dubois and Charpillet [25].

### 2.5. Data Analysis

First, we assessed how reliably our system could automatically measure TUG test duration. TUG test sequences were recorded for 37 participants. We assessed the level of agreement between the 99 durations obtained by healthcare professionals and those extracted automatically with the Kinect sensor. The agreement was tested using Bland–Altman 95% bias and limits of agreement (LoA), percentage error (PE), Spearman’s correlation, concordance correlation coefficient (CCC), intra-class correlation (ICC) and linear regression. The Bland–Altman analysis was adjusted for repeated measurements in a random-effects model [27]. The PE was calculated by dividing the limits of agreement by the mean of the values obtained with the two methods of measurement [28]. A bootstrap-method (with 2000 resamples) using bias corrected and accelerated (BCa) was used to estimate the 95% confidence interval of Spearman’s correlation. The CCC, adjusted for repeated measures [29], assessed the precision and deviation of data from the line of identity. The ICC [30] was used to assess absolute agreement. Finally, slope and goodness of fit were computed using linear regression. The normal distribution of the data was systematically tested using the Shapiro–Wilk test.

Next, we tried to identify the test parameters allowing us to best discriminate low vs. high fall risk individuals. These parameters were meant to complete the evaluation of the healthcare professionals using information they do not have access to when conducting the traditional version of the test. Participants were classified using the best time of their three trials, as is usually done by healthcare professionals, the best performance being considered as a good indicator of an individual’s capacities. The participants who performed the test in less than 13.5 s (eleven participants in our study) were considered as having a low risk of fall, whereas the participants who needed 13.5 s or more (the twenty six other participants) were considered as having a high risk of fall. In our study, we used 13.5 s as threshold because it was the threshold used by the clinicians at the institute where we performed the TUG test. Importantly, supplementary data analysis performed as control revealed that cut-off thresholds set between 13 s and 16.5 s did not alter the classification results. We tested simultaneously the 21 parameters described in Section 2.4 using a multivariate analysis of variance (MANOVA) with two groups, high and low fall risk. For each parameter, the difference between the two groups was analyzed using either a Student *t*-test or a Wilcoxon Rank Sum test (when the distribution of values deviated from normality or when variance was not homogeneous between groups).

Finally, participants were classified into three groups, based on the evaluation grid used by healthcare professionals. Thirteen participants were in class 0 (i.e., grid score of 0, no risk of fall), five participants were in class 1 (grid score of −1 or −2, moderate risk of fall), and sixteen participants were in class 2 (grid score inferior to −3, rehabilitation required). We then used supervised machine learning methods to predict the class of the thirty four participants. We generated all combinations of one to three features from the parameters described in Section 2.4 (such as mean and median step length, gait speed, time to sit down, etc). Each combination was evaluated using state-of-the-art classifiers. We used the scikit-learn implementation [31] of Nearest Neighbors, Linear Support Vector Machines (SVM), Radial Basis Function (RBF) SVM, Gaussian Process, Decision Tree, Random Forest, Neural Net, AdaBoost, Naive Bayes, Quadratic Discriminant Analysis (QDA) and the k-fold validation procedure to compare the classification performances based on the different subsets of features. The k-fold cross validation procedure was performed by dividing the database in 34 partitions corresponding to the 34 participants so that each participant could never be simultaneously in the testing and training set. The classification and score prediction was performed on the same trial (best one) as the one used by the healthcare professional to perform the grid evaluation.

## 3. Results

### 3.1. Reliability of the Sensor to Measure the Global Time of TUG

**Figure 4:**
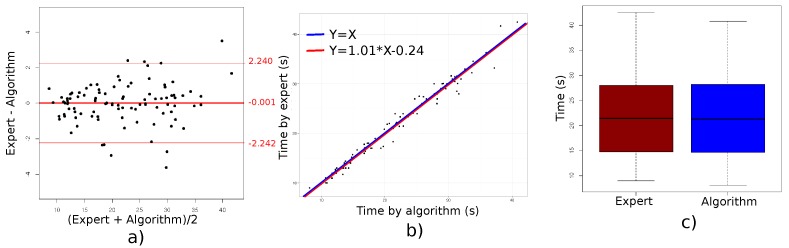
Different representations of the duration parameter. (**a**): Bland and Altman plots with three values by participants corresponding to the test duration according to the clinicians and to our algorithm; (**b**): scatter plots of all the data with red lines indicating the identity line and blue line corresponding to the linear best-fit; (**c**): boxplot showing the median and variability of the recorded data.

Table 1 reports the means and related standard deviations of test durations measured with the two methods (Kinect sensor algorithm and healthcare professional). Each duration was obtained for one sequence, so that we calculated the mean and standard deviation from 99 sequences. Overall, duration measurements obtained by clinicians and algorithm were very similar, as shown in Figure 4c).

The level of agreement between the algorithm and clinicians measurements was evaluated. Figure 4a shows the Bland and Altman plot for test durations. The mean difference (bias) and the LoA (mean differences ± 2 Standard Deviation (SD)) between test durations estimated by clinicians and our algorithm are shown in Table 2. Clinicians and algorithm provided very similar values (difference of −0.001 s). Moreover, as shown in Figure 4a, most of the points are within the interval given by the LoA. The 95% confidence interval was not reported here because the data was not normally distributed. Finally, the percentage of error (PE) was less than 10.5%.

Table 3 reports additional agreement parameters from Spearman, CCC and ICC measures. The agreement for the duration obtained by clinicians and our algorithm are equal to 0.99, which is excellent. The confidence interval of Spearman’s correlation is above 0.98. Figure 4b shows the data for clinicians and algorithm, and a comparison between the identity line and the linear best-fit. The slope of the regression line was equal to 1.01, indicating an excellent correspondence between the data provided by the algorithm and that provided by the clinicians.

### 3.2. Relevant Parameters to Discriminate Low and High Risk of Fall

The MANOVA indicated a significant effect of the class (“Low risk of fall” vs. “High risk of fall”) on the different parameters, F(21, 82) = 20, *p* < 0.001.

Regarding the results of the Student *t*-tests and Wilcoxon Rank Sum tests presented in Table 4, all *p*-values were below 0.05, indicating that, for all parameters, the values recorded in the “High risk of fall” group were significantly different from those recorded in the “Low risk of fall” group.

### 3.3. Prediction of the Grid Class

We used machine learning algorithms to test if we could correctly classify all participants in the same class as the clinical staff, who used the evaluation grid for the classification. We tested the classifiers with one to three parameters considering that more than three parameters could lead to over-fitting. Each model was evaluated using the k-fold cross validation score representing the rate of correct classification. The results are shown in Table 5. This table shows the parameters having the best rate of correct classification. The best possible score is 1. We can see that, with three parameters, the rate of correct classification was 94%. The gait parameters were used in the majority of the models giving the best results. Some best combinations of three parameters include parameters providing information about the quality of the transition from standing to sitting. The Neural Net, Naive Bayes, QDA and Decision Tree algorithms used to classify the data appeared more effective than the others algorithms.

We studied the error cases with the best models based on three parameters. We saw that these models committed only two errors out of the 34 sequences. Specifically, in some cases, two participants of class 1 (“moderate risk of fall”) were incorrectly classified in class 2 (“high risk of fall, needs a rehabilitation of the lower limbs”), whereas, in other cases, one participant of class 1 was incorrectly classified in class 2 and one participant of class 2 incorrectly classified in class 1. One likely explanation for these few classification errors between class 1 and 2 is that only a few participants were representative of class 1 (five participants in class 1 versus thirteen in class 0 and sixteen in class 2). Therefore, sensibility was lower for class 1 than for the other two classes. For class 0, sensibility and specificity scores both reached the maximum value 1. For classes 1 and 2, sensibility and specificity depended on the chosen model. For class 2, sensibility and specificity scores were 0.94 when one class 2 subject was incorrectly classified and 1 and 0.89, respectively, when all class 2 subjects were correctly classified. For class 1, the sensibility and specificity scores were 0.6 and 1, respectively, when two class 1 subjects were incorrectly classified and 0.8 and 0.96, respectively, when one class 1 subject was incorrectly classified.

No participant of class 1 or 2 was ever classified in class 0 (having no risk of fall), and no participant of class 0 was classified in class 1 or 2, what would have been the most critical errors. Considering that the most important issue was to be able to discriminate individuals with no risk of fall from individuals with low and high risk of fall, we also tried to classify the participants into two groups instead of three, by regrouping the participants of class 1 with those of class 2. The results for the classification in two groups are shown in Table 6. We can see that, with only two parameters, the algorithm provided a model that classified all participants in the correct class without error, the classification performed by the clinicians being used as a reference. The perfect model with two and three parameters coupled gait parameters and parameters related to the sitting position or the turn.

## 4. Discussion and Conclusions

Our aim was to automate the Timed Up and Go test in order to discriminate low vs. high risk of fall individuals as objectively as possible using several quantitative parameters. Currently, healthcare professionals time the test and can use an evaluation grid to perform the test more objectively. However, this test is often criticized because it depends too heavily on the clinician conducting it and on the environment in which it is performed. To automate the duration measurement and the evaluation grid, 37 individuals performed the TUG test in front of an ambient Kinect sensor. The sequences were simultaneously evaluated by healthcare professionals and by specific algorithms analysing the depth images provided by the Kinect sensor. The clinicians measured the total duration of the test and filled the evaluation grid for each participant. With our algorithms, we automatically measured the test duration as well as other parameters (such as step length, time to turn, etc) inspired by the evaluation grid.

The first important result is that we found an excellent agreement between the TUG test durations measured by the clinicians and those provided by our algorithm, with a difference in average duration of only −0.001 s. As a comparison, in a study with nine participants (including five elderly people), Lohmann et al. [22] found an average difference of 0.1 s when comparing Kinect-based and clinician-based measurements of test duration. In addition to assessing the agreement for average durations, we also compared algorithm-measured and clinician-measured test duration values for each of the 99 individual tests and computed the limits of agreement (mean difference ± 2 SD). The observed limits of agreement ranged from −2.24 to 2.24. Such a 2 s difference is rather small, especially considering that this difference depends on when the clinician starts the chronometer. As a matter of fact, measured durations can also differ between clinicians [32]. In our experiment, two clinicians timed the TUG test. We ran an extra analysis in which the durations measured by the two clinicians were analyzed separately using in each case the same statistical analysis that was used for the “global analysis”, i.e., the analysis that included the test durations measured by both clinicians. Test durations measured by clinician 1 were closer to those measured by the Kinect (limits of agreement ranging from −2.313 to 1.977 with a mean difference of −0.168) than test durations measured by clinician 2 (limits of agreement ranging from −2.185 to 2.584 with a mean difference of 0.199). Murphy and Lowe [32] compared test durations measured by two different clinicians on the same thirty test sequences (15 participants, two test sequences per participant). These authors did not report the limits of agreement (for a direct comparison with ours), but they found a correlation coefficient of 0.77 between the test duration measured by the two clinicians, which highlights some measurement differences. Taken together, these results confirm that our algorithm provides reliable measurements of the TUG test duration, which are in good agreement with those measured by expert clinicians.

A second objective of our study was to identify additional parameters (i.e., other than test duration) that are currently neither measured nor measurable by expert clinicians and that could help discriminate high and low fall risk individuals. Based on test duration, we classified our 37 participants into two groups, namely high fall risk and low fall risk individuals. The mean age of the participants in the high fall risk group was 86.63, whereas it was 79.36 in the low fall risk group. Interestingly, all automatically extracted parameters, such as step length, gait speed, time and speed to sit down and to get up, or time to walk and turn, differed significantly between the two groups. Previous studies tried to identify parameters other than test duration that could discriminate high and low fall risk individuals. For instance, Tmaura et al. [18] investigated each phase of the TUG test (i.e., sit-bend, bend-stand, forth walk, turn, back walk, stand-bend and bend-sit) using wireless inertia sensors. They showed that the duration required to complete each phase was significantly different between high fall risk and low fall risk individuals. Our results are similar to theirs. Therefore, these parameters could be used to complete the evaluation of healthcare professionals by providing complementary information. As proposed by Tmaura et al., these parameters would notably be very helpful in determining more precisely which activities would place the individual at a higher risk of fall and should therefore be monitored with particular attention. In addition, clinicians could rely on these parameters to propose a more targeted rehabilitation focusing particularly on the specific “weaknesses” of each patient.

The third objective of our study was to assess how well the parameters automatically extracted by our algorithm would allow us to classify participants into the three fall risk classes routinely used by clinicians. The classification performed by expert clinicians on our 34 participants was used as a reference. For a three-class classification, the best matching rate between our algorithm and the reference was 94%. This matching rate was obtained combining three evaluation parameters. By regrouping the two classes being composed of individuals at risk of fall (i.e., moderate and high risk of fall) so as to use a two-class classification, we obtained a matching rate of 100% using two evaluation parameters only. In other words, two parameters were sufficient to distinguish without error participants being at risk of fall from participants free of fall risk. Based on our results, the most relevant parameters to perform such a classification are mean step length, mean step duration and mean walking cadence. In particular, we showed that the most robust classification is obtained when coupling these parameters with parameters related to the sitting position (speed to sit down or to get up).

Because of population ageing, fall prevention is a very important issue, both nowadays and for years to come. People presenting a fall risk need to be detected as soon as possible so that they can adapt their home environment and follow rehabilitation programs allowing them to stay active longer, if possible at home. Therefore, fall risk represents a human, economic and societal issue. Currently, fall prevention mostly relies on clinical tests, and notably the TUG test, performed in healthcare institutions by expert clinicians. Here, we proposed a system that automatically evaluates TUG test performance in a more objective, robust and detailed manner. This system is inexpensive, easy to set up, and does not require the level of expertise of the traditional TUG test to be reliably used. Therefore, our system could be used routinely and with more flexibility by physicians/clinicians with less expertise (as, for instance, by general practitioners in their practice) to assess fall risk, which would greatly contribute to improving fall risk detection.

## Figures and Tables

**Table 1:** Duration statistics (s) of 99 measures obtained by clinicians and algorithm.

	Expert	Algorithm
Mean	21.723	21.722
Standard deviation	7.815	7.978
Min	8.17	8.99
Max	40.8	42.48

**Table 2:** Bland–Altman bias (limits of agreement), and percentage error (PE) (computed as 100 × (2 SD of bias)/(($Mean_{Algorithm}+Mean_{Expert}$)/2) for the two measurement types.

	Bias (95 % LoA)	PE (%)
Duration (s)	−0.001 (−2.242 to 2.240)	10.32

**Table 3:** Agreement computed with Spearman correlation (95% confidence intervals), concordance correlation coefficient (CCC) and intra-class correlation (ICC) analysis.

	Spearman (Intervals)	CCC	ICC
Duration	0.99 (0.98 to 0.99)	0.99	0.99

**Table 4:** Statistically significant differences between the “At risk of fall” (≥13.5 s on the TUG test) and the “Low risk of fall” (<13.5 s on the TUG test) group for automatically extracted parameters.

	Low Risk of Fall Mean ± Std	At Risk of Fall Mean ± Std	W or *t*	*p*-Value
TUG duration (s)	12.87 ± 2.12	25.78 ± 5.92	9	<0.001
Time to get up (s)	1.05 ± 0.35	2.50 ± 1.27	170.5	<0.001
Speed to get up (mm/s)	567.80 ± 127.26	363.46 ± 104.14	2065	<0.001
Time to sit down (s)	0.98 ± 0.59	3.99 ± 2.54	123.5	<0.001
Speed to sit down (mm/s)	−485.07 ± 139.44	−221.34 ± 85.78	83	<0.001
Time to walk (s)	10.84 ± 1.84	19.17 ± 4.22	38	<0.001
Number of steps (s)	6.87 ± 1.84	13.51 ± 3.39	71	<0.001
Greatest width of walk (cm)	612.14 ± 410.95	813.44 ± 400.68	791	0.016
Mean step length (cm)	48.93 ± 5.34	32.81 ± 5.58	2232	<0.001
Median step length (cm)	49.33 ± 5.57	33.38 ± 5.84	*t* = 12.908	<0.001
CV of mean step length (%)	11.63 ± 7.99	21.85 ± 8.71	328	<0.001
Mean step duration (s)	0.61 ± 0.062	0.73 ± 0.09	264	<0.001
Median step duration (s)	0.60 ± 0.06	0.72 ± 0.086	255.5	<0.001
CV of mean step duration (%)	10.51 ± 5.53	20.01 ± 6.83	297	<0.001
Mean cadence (steps/min)	1.68 ± 0.16	1.45 ± 0.16	*t* = 6.60	<0.001
Median cadence (steps/min)	1.67 ± 0.15	1.41 ± 0.16	2008.5	<0.001
CV of mean cadence (%)	10.85 ± 7.40	20.89 ± 7.81	280	<0.001
Gait speed (cm/s)	81.58 ± 11.66	45.78 ± 8.59	2261	<0.001
Number of stops	0.45 ± 0.68	0.94 ± 0.88	763	0.005
Time of turn (s)	1.58 ± 0.45	2.99 ± 0.98	121	<0.001
Width of turn (cm)	387.54 ± 311.69	546.13 ± 280.18	835	0.035

**Table 5:** Best parameter combinations for the three classes problem. The best classification score is presented in fourth column. A score of 1 would be a perfect score, i.e., without any classification error. The third column indicates the algorithm giving the best result (NN = Neural Net, NB = Naive Bayes, DT = Decision Tree, QDA = Quadratic Discriminant Analysis).

Number of Parameters	Parameters	Algorithm	Classification Rate
1	- Number of steps	DT	0.882
2	- TUG duration, Number of steps	NN	0.912
	- Number of steps, Mean or Median duration	NN, NB, QDA	
	- Time to walk, Mean cadence	NB	
	- Number of steps, Mean cadence	DT	
	- Number of steps, Median cadence	NN	
3	- Number of steps, Mean duration or cadence, Speed to sit down	NN	0.941
	- Time to walk, Mean length, Mean or Median duration or cadence		
	- Number of steps, Mean or Median duration, Median length		
	- Number of steps, Mean cadence, Median length		
	- Time to walk, Number of steps, Median cadence		
	- Time to walk, Mean duration, Speed to sit down		
	- Number of steps, Mean cadence, Gait speed		
	- Time to walk, Median duration or cadence, Gait speed	NB	
	- TUG duration, Median cadence, Gait speed		

**Table 6:** Best parameter combinations for the two classes problem. The best classification score is presented in the fourth column. A score of 1 would be a perfect score, i.e., without any classification error. The third column indicates the algorithm giving the best result (NN = Neural Net, NB = Naive Bayes, DT = Decision Tree, Random Forest = RF, AB = AdaBoost, N = Nearest Neighbors, QDA = Quadratic Discriminant Analysis).

Number of Parameters	Parameters	Algorithm	Classification Rate
1	- Time to walk	DT, N, RF, NN	0.941
		AB, NB, QDA	
	- Gait speed	NN	
2	- Number of steps, Gait speed	QDA	1.0
3	- Time to walk, Mean duration, Speed to get up	N	1.0
	- Speed to sit down, Number of steps, Mean duration	N, NN	
	- Speed to sit down, Time to walk, Mean duration	QDA	
	- Time to get up or Number of steps, Mean length, Mean duration		
	- Speed to sit down, Number of steps, Mean cadence		
	- Number of steps, Mean length, Mean cadence		
	- Mean length, Mean duration, Mean cadence		
	- TUG duration, Number of steps, Gait speed		
	- Time to walk or to turn or to get up, Number of steps, Gait speed		
	- TUG duration, Mean duration or cadence, Gait speed		
	- Number of steps, Mean duration or cadence, Gait speed		
	- Time to get up, Mean duration, Gait speed		
	- Mean cadence, Gait speed, Time to turn	NB, QDA	
	- TUG duration, Number of steps, Mean duration	NN	
	- Time to sit down or to walk, Number of steps, Mean duration		
	- Number of steps, CV length, Mean duration		
	- TUG duration, Speed to get up, Mean cadence		
	- Speed to get up or TUG duration, Time to walk, Mean cadence		
	- Speed to get up, Number of steps, Mean cadence		
	- TUG duration, Mean duration, Gait speed		
	- Number of steps, Mean or median duration, Number of stop		
	- Number of step, Mean cadence or duration, Time to turn		
	- Mean length, Mean duration, Time to turn		
	- Time to get up, Median length, Mean duration	QDA, NN	
	- TUG duration, Time to walk, Mean cadence		
	- Time to get up, Mean duration, Gait speed	NB	
	- Time to get up, Mean or median cadence, Gait speed		
	- Mean cadence, Gait speed, Number of stop		
	- Time to walk, Mean cadence, Time to turn		
	- Median cadence, Gait speed, Time to turn		
	- Mean duration, Gait speed, Number of stop or Time to turn	NN, NB	
